# Prevalence of Chlamydial Infections in Fattening Pigs and Their Influencing Factors

**DOI:** 10.1371/journal.pone.0143576

**Published:** 2015-11-30

**Authors:** Karolin Hoffmann, Franziska Schott, Manuela Donati, Antonietta Di Francesco, Michael Hässig, Sabrina Wanninger, Xaver Sidler, Nicole Borel

**Affiliations:** 1 Institute of Veterinary Pathology, Vetsuisse Faculty, University of Zurich, Zurich, Switzerland; 2 Center for Clinical Studies, Vetsuisse Faculty, University of Zurich, Zurich, Switzerland; 3 Department of Farm Animals, Division of Swine Medicine, Vetsuisse Faculty, University of Zurich, Zurich, Switzerland; 4 DIMES, Microbiology, University of Bologna, Bologna, Italy; 5 Department of Veterinary Medical Sciences, University of Bologna, Ozzano Emilia (BO), Italy; 6 Department for Farm Animals, Section for Herd Health, Vetsuisse Faculty, University of Zurich, Zurich, Switzerland; University of the Pacific, UNITED STATES

## Abstract

Chlamydial infections in pigs are associated with respiratory disease, diarrhea, conjunctivitis and other pathologies. The aim of this study was to define the prevalence of *Chlamydiaceae* in Swiss fattening pigs by applying sensitive and specific detection methods and to correlate prior antibiotic treatment and farm related factors with differences in prevalence. Conjunctival and fecal swabs were collected from 636 pigs in 29 Swiss fattening pig farms with and without antibiotic treatment, at the beginning and the end of the fattening period. The swabs were screened by real-time PCR for *Chlamydiaceae*. For the chlamydial detection and species-identification, a DNA-microarray analysis was performed. All farms were positive for *Chlamydiaceae* with 94.3 and 92.0% prevalence in fecal swabs as well as 45.9 and 32.6% in conjunctival swabs at the first and second time points, respectively. Antibiotic treatment could not clear the infection on herd level. Potential contact with wild boars was a significant risk factor, while hygiene criteria did not influence chlamydial prevalence. A correlation of chlamydial positivity to diarrhea, but not to conjunctivitis was evident. *Chlamydia suis* was the predominant species. Mixed infections with *C*. *suis* and *C*. *pecorum* were common, with a substantial increase in *C*. *pecorum* positivity at the end of the fattening period, and this finding was associated with ruminant contact. *C*. *abortus* was detected in one conjunctival swab. In this study, *C*. *suis* inhabited the intestinal tract of nearly all examined pigs, implying a long-term infection. *C*. *pecorum* was also common and might be transmitted to pigs by ruminants.

## Introduction

Members of the *Chlamydiaceae* are known to cause a broad spectrum of diseases in numerous vertebrate host species worldwide. Chlamydiae are obligate intracellular bacteria sharing a characteristic biphasic lifecycle. Extracellular elementary bodies (EBs) infect the host cell and transform into the replicating reticulate bodies (RBs), which then re-differentiate into EBs. Replication occurs primarily in epithelial cells of the respiratory, gastrointestinal and urogenital tract as well as in the conjunctival epithelium [1].

To date, there are eleven species included in the Genus *Chlamydia*, four of which have been documented in pigs: *Chlamydia suis*, *Chlamydia pecorum*, *Chlamydia abortus* and *Chlamydia psittaci*, with *C*. *suis* being the most important. *Chlamydiaceae* in pigs are associated with disorders like conjunctivitis, pneumonia, pericarditis, polyarthritis, polyserositis, enteritis and reproductive problems of sows and boars; however, infections are often asymptomatic and routine diagnostics usually do not include chlamydiae [1]. The seropositivity for *Chlamydiaceae* in pigs from European countries is high and ranges up to 96.5% [2–7]. Not only domestic pigs but also wild boars (*Sus scrofa*) harbor *Chlamydiaceae*, as reported in Germany, Spain and Italy [8–12]. In fecal samples from pigs, *C*. *suis* is the most frequently found chlamydial species [7,13]. Although *C*. *suis* is most often found in pigs, many other animal species like cattle, horses, frogs and cats can become naturally infected [14–17]. Evidence for the zoonotic potential of *C*. *suis* is emerging, as previously demonstrated in trachoma patients from Nepal, where *C*. *suis* was found as a single or mixed infection with *C*. *trachomatis* [18]. Moreover, *C*. *suis* DNA was found in conjunctival swabs of employees in a Belgian pig slaughterhouse [19,20].

In Switzerland, prophylactic and metaphylactic antimicrobial treatment of fattening pigs is often applied, mostly with sulfonamide and trimethoprim combinations or tetracyclines [21,22]. For decades, tetracyclines, as broad-spectrum antibiotics, have been extensively used in the pig industry for both prophylactic and therapeutic treatment. Over the past years, there have been accumulating reports on the occurrence of tetracycline-resistant *C*. *suis* strains in America, Europe and Asia [23–27]. A rapid selection for tetracycline-resistant *C*. *suis* was detected in a Swiss farm after oral tetracycline medication, while elimination of the organism was not achieved [26].

In Switzerland, pigs are the most important source of meat for human consumption [28] and therefore the pig producing industry plays an important role in human nutrition and the agricultural economy. Consequently, pig pathogens potentially play an important role in human public health.

To date, no large-scale prevalence studies on *Chlamydiaceae* in pigs exist. The available data were obtained by serological methods and included mostly breeding farms or breeding sows [1]. The aim of this study was to collect comprehensive data by applying sensitive and specific direct detection methods to determine the prevalence of infection with all currently known *Chlamydiaceae* species in the Swiss fattening pig population. Results were correlated with medical treatment, hygiene and housing conditions, as well as contact with other farm and/or wild animal species.

## Material and Methods

The prevalence of *Chlamydiaceae* in Swiss fattening pigs was investigated by sampling conjunctival and fecal swabs from 29 different pig herds at the beginning and end of the fattening period.

### Farms and animals

In total, 1,359,513 pigs, excluding breeding sows, lived in Switzerland as of May 1^st^, 2013 [29] and 2,689,327 pigs were slaughtered in the year 2013 [28].

A total of 29 fattening pig farms (S1 Table) were included in this study and were sampled between September 2013 and December 2014. They were geographically located in the main farm animal producing lowlands of the central part of Switzerland, comprising nine Swiss cantons (Aargau: n = 3, Bern: n = 3, Freiburg: n = 1, Luzern: n = 12, St. Gallen: n = 2, Schaffhausen: n = 1, Thurgau: n = 2, Waadt: n = 2, Zürich: n = 3). The cantons examined harbor the following proportions of the Swiss pig population [29]: Aargau: 6.5%, Bern: 17.1%, Freiburg: 5.5%, Luzern: 27.6%, St. Gallen: 12.2%, Schaffhausen: 1.3%, Thurgau: 12.6%, Waadt: 2.9%, Zürich: 2.6%, thus, the most important pig producing regions were covered by the sampling. Farm participation was confirmed upon the request of the farm veterinarians (n = 12), the two main Swiss pig-trading companies (n = 10), the Swiss Pig Health Service (n = 2) or directly by the study investigator (n = 5). The study was approved by the Veterinary Office of Canton Luzern (authorization no. LU03/14) and all efforts were made to minimize the discomfort of the animals during sampling. The pig housings and land that was accessed are privately owned and all owners gave their permission to conduct the study on these sites.

The farms were divided into two groups: farrow-to-finish farms (n = 10) and fattening farms without breeding on farm (n = 19). An all-in/all-out-production system was used on seven farms, while nine out of ten farrow-to-finish farms and 13 fattening farms were rearing the pigs in a continuous system. Regarding the housing system, there were farms with (n = 19) and without (n = 10) outdoor access combined with indoor batch pens. The floor of the indoor pens was covered with bedding material in most farms (n = 19). Outdoor access and presence of bedding material in indoor pens was often combined on the same farm (n = 16). Eight farms routinely applied prophylactic oral antibiotic treatment of the whole herd (combination of trimethoprime, sulfadimidin and sulfathiazole (TSS) on farms 6, 23 and 26; combination of chlortetracycline, tylosin and sulfadimidin on farms 9, 22, 27 and 29; chlortetracycline in farm 17), whereas one farm administered amoxicillin (A) therapeutic group treatment at the beginning of the fattening period (farm 7). On one farm, a combination of chlortetracycline, sulfadimidine and tylosin was known to be administered to the pigs before their entry into the fattening period (farm 28). All group treatments were administered orally for five to twelve days. Individual medical treatment was carried out on 15 farms, nine farms did not administer any medication during the whole sampling period and on farm 8 no medication data was available (S1 Table).

In farm nos. 1, 5 and 9, all newly restocked fattening pigs in the herd were sampled, whereas in the remaining farms (n = 26), 20 randomly selected pigs per newly restocked pig batch were sampled regardless of herd size. In total, 636 pigs were included in this study. Twenty-seven farms reared male and female fattening pigs, two farms (farm nos. 18 and 25) were farrow-to-finish pig farms that only fattened female pigs. Overall, 54.7% (n = 348) of the pigs were female and 45.3% (n = 288) were male. Identical pigs were sampled twice (n = 589): i) at the beginning (first sampling, 1^st^) of the fattening period (at the age of approximately 12 weeks), and ii) at the end (second sampling, 2^nd^) of the fattening period (at the age of approximately six months). The first sampling was performed as early as possible after the pigs were introduced into their new pens and before any medical treatment was started, which was between 0 and 72 hours after their arrival. The second sampling was performed approximately one to three weeks prior to slaughtering. In 47 pigs, a second sampling was not possible. In one farm (no. 10), porcine reproductive and respiratory syndrome virus was detected after the first sampling procedure and led to the subsequent eradication of all pigs on this farm and a second farm (no. 8) was lost to follow-up. In another six farms, one animal each (farm nos. 5, 6, 11, 12, 13) and two animals (no. 14), respectively, could not be sampled a second time, because they had been slaughtered or died spontaneously during the fattening period.

### Swab sampling

Conjunctival and fecal flocked swabs (FLOQSwabs®, Copan Italia, Brescia, Italy) from fattening pigs (n = 636 at first time point, n = 589 at second time point) were collected between September 2013 and December 2014. Both eyes of each individual pig were sampled using one conjunctival swab. Fecal swabs were taken by rectal insertion of flocked swabs. Additionally, conjunctival swabs from voluntarily participating farmers (n = 9) were obtained with their written informed consent of seven different farms, and swabs (n = 2) from dust in the housing environment of farm no. 29 were collected. A total of 2,461 swabs were obtained and stored at -20°C until processing.

### Questionnaire and health assessment

A detailed questionnaire was obtained from the farmers and a summary of the parameters is shown in S2 Table. The cleanliness of the sampled animals and the facilities (housing in general, indoor pens, outdoor area when existent) were recorded by the study investigators at both time points and classified as clean, moderately dirty or dirty. Hygiene management was scored by giving one point each for the following hygiene criteria as reported by the farmer: Existence of hygiene gate to pig housings, cleaning of pens before introducing new pigs, water temperature in high-pressure cleaner at least 60°C, use of cleaning agents and use of disinfectant on a regular basis. With five points as the maximal score, four to five points were regarded as most hygienic, two to three points as medium and zero to one point as least hygienic. Recruitment of animals was scored as follows: 0 equals recruitment exclusively from own farm (n = 9), 1 equals purchase from one farm (n = 9), 2 equals purchase from 2 to 4 farms (n = 8) and 3 equals purchase from more than 4 farms (n = 3). All pig herds had at least one ruminant herd in their surrounding area of up to 1000 meters, belonging to the same or neighboring farms. The distance to ruminants was scored as follows: 1 equals direct snout contact is possible (n = 5, S1 Table), 2 equals one to ten meters distance (n = 12), 3 equals 11 to 100 meters distance (n = 7) and 4 equals 150 to 1000 meters distance (n = 5). Indirect contact with ruminants was defined as potential transmission routes by personnel and non-living vectors, such as instruments used in ruminant and pig housings, and occurred in 17 farms. Poultry was present within a 1000 m radius of 19 farms and in eight of these the birds belonged to the same farm. In none of the 19 farms was direct contact between poultry and fattening pigs possible. Direct contact with wild boars and other wild mammals like roe deer was possible due to the absence of double enclosures in 9 farms, and 25 herds had potential contact with wild birds. Insect and rodent infestation was estimated by the farmers and graded as low, moderate or high. The farmers were asked to record clinical symptoms and any treatment of the sampled pigs during the fattening period. The health status of the sampled pigs was assessed on the occurrence of eye lesions, change in fecal consistency, respiratory signs, wasting and lameness by the study investigators. Eye changes were assessed in accordance with Englund et al. [30], Polkinghorne et al. [31] and Becker et al. [32] and included reddening (hyperemia) and swelling (edema) of the conjunctiva and/or sclera, epiphora, as well as mucous or purulent discharge.

### DNA extraction

DNA from all conjunctival and fecal swabs was extracted using the Maxwell® 16 Buccal Swab LEV DNA Purification Kit (Promega, Madison, WI, USA) according to the manufacturer’s instructions with an elution volume of 100 μl.

### Screening for *Chlamydiaceae*

All samples (n = 2,461) were examined using a 23S rRNA gene-based *Chlamydiaceae* family-specific real-time PCR with an internal amplification control as described previously by Blumer et al. [33] on an ABI 7500 instrument (Applied Biosystems, Foster City, CA, USA). All samples were tested in duplicate with a cycle threshold value set at 0.1 in each run. A mean cycle threshold (Ct value) of < 38 was considered positive. The corresponding *Chlamydiaceae* copy number per μl was automatically calculated by the PCR instrument for each tested sample. In cases of an inhibited amplification of the internal control DNA, the run was repeated with a 1:10 dilution of the sample. A sevenfold dilution series of *C*. *abortus* DNA constituting the standard curve served as positive control and a reaction mixture with water instead of the template DNA was used as negative control.

### *Chlamydia* species identification by Arraymate microarray

All samples classified as positive by real-time PCR (mean Ct value of < 38) were further examined by the species-specific 23S Arraymate microarray assay (Alere, Jena, Germany), as described by Borel et al. [34]. The present version [35] carries 34 probes for eleven species of *Chlamydiaceae* (four probes each for *C*. *suis*, *C*. *trachomatis*, *C*. *pneumoniae* and *C*. *psittaci*, three probes for *C*. *avium*, *C*. *caviae*, *C*. *muridarum* and *C*. *pecorum*, two for *C*. *abortus*, *C*. *felis* and *C*. *gallinacea*), three genus-specific probes, four family markers and 15 probes for *Chlamydia*-like organisms, as well as four probes for the internal control DNA and an internal staining control (biotin marker). Sample DNA was amplified and biotin labeled prior to hybridization according to Borel et al. [34], with the following temperature-time profile: 96°C 10 min, 40 cycles of 94°C 30 s, 50°C 30 s, 72°C 30 s, additionally an internal control DNA was included as recommended by the manufacturer (Intype IC-DNA, Qiagen Labor, Leipzig). In the first step, 8 μl of amplification product was loaded on the chip. The *Chlamydia* species could not be determined when only genus-specific probes were positive and the species-specific probes produced no or only a weak signal.

### Data analysis

Data editing and all statistical analyses were done using Stata Software (StataCorp., 2011; Stata Statistical Software: Release 12; College Station, TX, USA: StataCorp LP). Firstly, a quality control of the data and the descriptive analysis was carried out using <codebook varx1 varxn>, where varx1 to varxn represents the variables from x_1_ to x_n_. The dependence of a positive result in the conjunctival and rectal swab in pigs at one time point and between the two time points was analysed using the chi-square-test. To identify the influencing factors on positivity in conjunctival and fecal swabs at either time point for *Chlamydiaceae*, *C*. *suis* and *C*. *pecorum*, a logistic regression analysis for the independent variables sex, type of farm (farrow-to-finish or fattening), all-in/all-out production or continuous production, number of purchase farms, total number of fattening pigs on farm, outdoor access, existence of bedding material, existence of own or foreign ruminant species, possible direct or indirect contact and distance to ruminants, possible contact to wild boars or wild birds, clinical symptoms (conjunctivitis score; diarrhea, coughing, lameness of the group as reported by farmer), hygiene score, infestation with flies and rodents, cleanliness of animals and facilities at first and second time point and the use and type of antibiotics was carried out using <logistic vary varx1 varxn>, where vary represents the dependent variable, varx1 to varxn represents the dependent variable from x_1_ to x_n_. The significantly influencing variables were tested for their correlation using the <pwcorr varx1 varxn> command. If correlations of > 0.6 occurred, one of the variables was eliminated. The remaining variables were entered into a full regression model for step back procedure [36]. The final model included again only significant dependent variables. The mean *Chlamydiaceae* copy numbers per μl of extracted DNA for all positive samples were calculated separately for conjunctival and fecal swabs from the first and second time point, respectively. Additionally, an analysis of variance was carried out with the *Chlamydiaceae* copy numbers of all samples in regard of anatomical site, time point and antibiotic group treatment. In all analyses, a p-value ≤ 0.05 was considered statistically significant.

## Results

### Clinical symptoms

Six farmers stated that they had observed diarrhea during the fattening period. The following infectious agents could be detected by further laboratory investigations initiated by the respective farm veterinarians: *Brachyspira pilosicoli* and *Lawsonia intracellularis* in farm 4, *Brachyspira hyodysenteriae* in farm 9 and *Lawsonia sp*. in farm 19. Farms 11, 17 and 26 had only rare cases of diarrhea, of which infectious agents were not further investigated. In one additional farm (no. 18), the farmer did not report diarrhea, but clinical symptoms were visible in the majority of pig pens at both time points during farm visits by the study investigators.

Sixteen farmers observed lameness in one or more pigs during the fattening period. A diagnostic workup for infectious diseases was not performed in any of these cases, most farmers suspected *Haemophilus parasuis* and/or tail biting (cannibalism) as the cause, one farm (no. 12) assumed a broken leg, and another (no. 4) claw problems (panaritia).

In eleven farms cases of respiratory disease were observed by the farmers, but except for two farms (farm nos. 4 and 7), in which influenza was diagnosed, no causative agents were investigated.

One farm was known to have conjunctivitis problems in its finishing pigs. In another farm unclassified eye symptoms were observed during the fattening period; all other farmers reported no eye problems. The results of the conjunctivitis assessment in combination with conjunctival chlamydial positivity are displayed in Table 1. While the conjunctival lesions were significantly increasing, the chlamydial detection in conjunctival swabs significantly decreased between the first and second time point.

**Table 1:** Presence of eye lesions and chlamydial positivity by *Chlamydiaceae* specific real-time PCR. 1^st^ = sampling at the beginning of the fattening period; 2^nd^ = sampling at the end of the fattening period.

Eye lesions	No. animals 1^st^ (%)	No. chl.-pos. animals ^a^ ^)^ (%)	No. animals 2^nd^ (%)	No. chl.-pos. animals ^a^ ^)^ (%)
**No**	572 (89.9)	274 (93.8)	369 (62.6)	126 (65.6)
**Yes**	64 (10.1)	18 (6.2)	220 (37.4)	66 (34.4)
**Total**	**636 (100)**	**292 (100)**	**589 (100)**	**192 (100)**

^a^ positive by *Chlamydiaceae* specific real-time PCR.

None of the farmers reported herd problems of wasting, however, there were a few pigs (n = 7) at the first sampling and one pig at the second sampling that were considerably retarded in growth.

### *Chlamydiaceae* screening

Pigs from all farms were positive for *Chlamydiaceae* at both samplings (S3 Table, Table 2). In the first sampling, 45.9% (n = 292) and in the second sampling 32.6% (n = 192) of the pigs were positive in the conjunctival swab. The herd-based prevalence on conjunctival swabs ranged from 0 to 100% with a mean value of 38.7%. In contrast, 94.3% (n = 600) of the fecal swabs were positive in the first sampling and 92.0% (n = 542) in the second sampling. The herd prevalence ranged from 55 to 100% with a mean value of 93.0%. Only one pig from farm 18 was negative in all samples, it had diarrhea of unknown origin at both samplings and mild conjunctival reddening at the second sampling. Another six pigs were questionably positive in at least one of the samples taken and negative in the remaining samples, resulting in a total of 98.9% (n = 629) *Chlamydiaceae* positive pigs in at least one sample. On the other hand, 12.7% of the pigs (n = 81) were positive in all samples taken. In one farm (no. 29), two dust swabs taken from the housing environment were both positive for *Chlamydiaceae*.

**Table 2:** Results of *Chlamydiaceae* screening according to antibiotic group treatment.

farm	Group treatment ^a)^	conjunctival swab			fecal swab		
		No. of pigs positive at 1^st^ sampling (%)	No. of pigs positive at 2^nd^ sampling (%)	Difference 1^st^ to 2^nd^ (%)	No. of pigs positive at 1^st^ sampling (%)	No. of pigs positive at 2^nd^ sampling (%)	Difference 1^st^ to 2^nd^ (%)
1–5, 8, 10–16, 18–21, 24, 25, 28	None	221/440 (50.2)	146/394 (37.1)	-13.1	421/440 (95.7)	379/394 (96.2)	+0.5
9, 17, 22, 27, 29	C or CST	41/116 (35.3)	30/116 (25.9)	-9.4	104/116 (89.7)	94/116 (81.0)	-8.7
6, 23, 26	TSS	24/60 (40.0)	16/59 (27.1)	-12.9	57/60 (95.0)	50/59 (84.7)	-10.3
7	A	6/20 (30)	0/20 (0)	-30	18/20 (90)	19/20 (95)	+5
**total**		**292/636 (45.9)**	**192/589 (32.6)**	**-13.3**	**600/636 (94.3)**	**542/589 (92.0)**	**-2.3**

^a)^ pro-/metaphylactic group treatment after 1^st^ sampling, type of antimicrobial substance: A = amoxicillin; C = chlortetracycline; CST = chlortetracycline, sulfadimidin, tylosin; TSS = trimethoprime, sulfadimidin, sulfathiazole. 1^st^ = sampling at the beginning of the fattening period; 2^nd^ = sampling at the end of the fattening period.

Regardless of the sampling time point, individual pigs were most frequently positive in fecal and negative in conjunctival swabs (312/636 pigs at first sampling, 356/589 pigs at second sampling). The second most common finding was a positive conjunctival and fecal swab in individual pigs (288/636 pigs at the first sampling and 186/589 at the second sampling).

A majority of pigs (n = 513) were positive at both samplings in fecal swabs. In addition, pigs (n = 36) negative in fecal swabs at the first sampling showed a significant change to positive at the second sampling; specifically, 80.6% (n = 29) became positive at the second sampling and 19.4% (n = 7) remained negative. A small proportion of the positive fecal samples from the first sampling became negative in the second (7.2%, n = 40). Comparing the first and second sampling, *Chlamydiaceae* prevalence rates in the conjunctival swabs were not significantly correlated to the sampling time point.

The mean *Chlamydiaceae* copy numbers per μl of extracted DNA for positive conjunctival swabs were 108 and 437 at the first and second time point, respectively. In fecal swabs, the mean of the positives were 3913 at the first, and 668 copies per μl at the second time point. Regarding all swab samples, the anatomical site and the time points were significant influencing factors, whereas antibiotic group treatment was not.

### *Chlamydia suis*

All farms were positive for *C*. *suis* at both sampling time points, as shown in Table 3. This chlamydial species accounted for the largest proportion of the *Chlamydiaceae* positive samples: 94.2% (n = 275) and 76.0% (n = 146) in the conjunctival swabs as well as 90.8% (n = 545) and 80.4% (n = 436) in the fecal swabs at both time points, respectively. Both dust swabs from farm no. 29 were *C*. *suis* positive. Mixed infections with *C*. *pecorum* (n = 85) and *C*. *abortus* (n = 1) were present (Table 3).

**Table 3:** Chlamydial species differentiation in *Chlamydiaceae* positive samples. 1^st^ sampling = sampling at the beginning of the fattening period; 2^nd^ sampling = sampling at the end of the fattening period.

species	conjunctival swab		fecal swab	
	No. of pigs tested positive at 1^st^ sampling (%)	No. of pigs tested positive at 2^nd^ sampling (%)	No. of pigs tested positive at 1^st^ sampling (%)	No. of pigs tested positive at 2^nd^ sampling (%)
***C*. *suis***	266 (91.2)	138 (71.9)	544 (90.6)	368 (67.9)
***C*. *pecorum***	1 (0.3)	1 (0.5)	0 (0)	13 (2.4)
***C*. *suis + C*. *pecorum***	8 (2.7)	8 (4.2)	1 (0.2)	68 (12.5)
***C*. *suis + C*. *abortus***	1 (0.3)	0 (0)	0 (0)	0 (0)
**Not determined**	16 (5.5)	45 (23.4)	55 (9.2)	93 (17.2)
**Total**	**292 (100)**	**192 (100)**	**600 (100)**	**542 (100)**

### *Chlamydia pecorum*

*C*. *pecorum* was detected in 15 farms at the second sampling and in three of these also at the first sampling (Table 3). In one of the farms (no. 29), *C*. *pecorum* was found in one of two dust swabs from the housing environment but not in swabs from tested pigs. In total, 14.5% (n = 92) of the pigs were infected with *C*. *pecorum* at least at one site and one sampling time point. Of nine animals (1.4%) positive in at least one site at the first sampling, four were females and five were males. Later on, of 86 animals (14.6%) positive at the second sampling, 59 were females and 27 were males. Three pigs were positive at least at one site at both time points, all others were only positive at one sampling time point (n = 89). The total number of conjunctival specimens positive at both time points did not change (n = 9) but was found in different pigs. In contrast, the positive fecal samples underwent a considerable increase between the first and second time point from one to 81 animals on 14 different farms (0.2% to 13.8%). Few pigs were positive for *C*. *pecorum* at both sampling sites, one pig at the first sampling and four pigs at the second sampling. *C*. *pecorum* infections were mostly mixed infections with *C*. *suis*.

### *Chlamydia abortus*

In farm 15, one animal at the first sampling was positive for *C*. *abortus* in the conjunctival swab (Table 3) and it was also positive for *C*. *suis* in the same sample. At the second sampling, the conjunctival swab of this animal was negative by means of PCR. The conjunctivae of this pig showed mild reddening at the first and moderate reddening at the second sampling, otherwise the pig appeared clinically healthy. The fecal swabs of this pig were positive at both time points, however, the Arraymate could not identify the chlamydial species from the fecal swab of the first sampling, but revealed *C*. *pecorum* in the second swab. These results indicate a potential mixed infection of this pig with *C*. *abortus*, *C*. *pecorum* and *C*. *suis*. On this farm no prophylactic, metaphylactic, therapeutic antibiotics or other medications were administered. Strikingly, it was one of two farms with direct contact to sheep in the outdoor area. The first sampling was performed three days after the pigs’ arrival at the farm.

### Human samples

All conjunctival samples obtained from farmers (n = 9) were negative by the *Chlamydiaceae*-specific PCR.

### Clinical symptoms and influencing factors

There was no correlation between the presence of conjunctivitis and chlamydial positivity in the conjunctival swabs. In farms with diarrhea observed by the farmers, the likelihood of the individual animals having a chlamydial infection was higher compared to that of other farms (Table 4). Lameness observed by the farmers was also positively correlated to *Chlamydiaceae* positivity at both time points (Table 4). In contrast, there was no association between respiratory signs, wasting and chlamydial prevalence.

**Table 4:** Results of the univariate logistic regression for clinical symptoms and influencing factors.

Independent variable	Dependent variable ^a)^	Time point	OR	CI (95%)
**Diarrhea**	*Chlamydiaceae* ^*^	1^st^	11.1	1.5, 81.9
**Diarrhea**	*C*. *suis* ^*^	1^st^	1.8	1.1, 2.8
**Diarrhea**	*C*. *suis* ^*^	2^nd^	1.6	1.03, 2.6
**Diarrhea**	*C*. *pecorum* ^*^	2^nd^	2.7	1.7, 4.5
**Lameness**	*Chlamydiaceae*	1^st^	2.9	1.4, 6.0
**Lameness**	*Chlamydiaceae*	2^nd^	1.9	1.04, 3.4
**Farrow-to-finish farms**	*Chlamydiaceae*	2^nd^	0.3	0.2, 0.5
**Farrow-to-finish farms**	*C*. *suis*	2^nd^	0.4	0.3, 0.5
**Continuous production**	*Chlamydiaceae*	2^nd^	0.3	0.2, 0.6
**Continuous production**	*C*. *suis* ^*^	2^nd^	0.7	0.4, 0.96
**No. of purchase farms**	*Chlamydiaceae*	2^nd^	1.4	1.1, 1.8
**No. of purchase farms**	*C*. *suis*	2^nd^	1.3	1.1, 1.6
**Direct contact with ruminants**	*Chlamydiaceae*	2^nd^	3.5	1.4, 8.8
**Direct contact with ruminants**	*C*. *pecorum*	2^nd^	7.1	4.3, 11.7
**Indirect contact with ruminants**	*Chlamydiaceae*	2^nd^	2.9	1.7, 5.2
**Indirect contact with ruminants**	*C*. *suis*	2^nd^	2.8	1.7, 4.5
**Indirect contact with ruminants**	*C*. *pecorum*	2^nd^	3.9	1.4, 10.9
**Distance to ruminants**	*Chlamydiaceae*	2^nd^	0.6	0.4, 0.7
**Distance to ruminants**	*C*. *suis*	2^nd^	0.7	0.6, 0.9
**Distance to ruminants**	*C*. *pecorum*	2^nd^	0.5	0.4, 0.6
**Outdoor access**	*C*. *pecorum*	2^nd^	15.4	5.6, 42.6
**Bedding material**	*C*. *pecorum*	2^nd^	3.9	2.1, 7.0
**Potential direct contact with wild boars**	*Chlamydiaceae*	2^nd^	2.1	1.2, 3.8
**Potential direct contact with wild boars**	*C*. *suis*	2^nd^	1.9	1.2, 2.9
**Potential direct contact with wild boars**	*C*. *pecorum*	2^nd^	6.0	3.7, 9.8
**Cleanliness of housing facilities at first sampling**	*Chlamydiaceae*	2^nd^	2.5	1.3, 4.7
**Cleanliness of housing facilities at first sampling**	*C*. *suis*	2^nd^	2.0	1.3, 3.0
**Cleanliness of housing facilities at first sampling**	*C*. *pecorum*	2^nd^	11.3	3.0, 42.0
**Prophylactic antibiotic group treatment**	*Chlamydiaceae* *	1^st^ pos → 2^nd^ neg	4.7	2.4, 9.1
**Prophylactic antibiotic group treatment**	*Chlamydiaceae* *	1^st^ neg → 2^nd^ neg	13.9	1.7, 115.9

^a)^ animals positive in conjunctival and/or fecal swab.

*: fecal swab considered.

1^st^ = sampling at the beginning of the fattening period; 2^nd^ = sampling at the end of the fattening period; OR: odds ratio; CI: confidence interval.

In farrow-to-finish farms, the risk of chlamydial infection at the second sampling was lower than in fattening farms and a continuous production system had a lower risk of chlamydial infection than an all-in/all-out production system (Table 4). The risk of chlamydial infection at the second time point increased depending on the number of purchase farms. Direct or indirect contact to ruminants was a risk factor for a higher rate of chlamydial infections at the second sampling time point. Moreover, the distance to surrounding ruminants significantly influenced the positivity rate at the second sampling. An increasing distance between pigs and ruminants resulted in a decreased chlamydial infection risk (Table 4). In *C*. *pecorum*, outdoor access and the presence of bedding material had a significant influence on chlamydial positivity. Pigs reared in farms with potential direct contact to wild boars had a higher risk of being infected at the second sampling time point. The observed cleanliness of the housing facilities at the first sampling had an impact on the positivity at that time point: the dirtier the facilities were evaluated, the higher was the chlamydial prevalence (Table 4).

No clear association was found between chlamydial infection (*Chlamydiaceae*, *C*. *suis*) and the sex of the pig, the total number of fattening pigs on the farm, outdoor access and existence of bedding material (*Chlamydiaceae*, *C*. *suis*), contact to wild birds, the hygiene score and the level of infestation with insects or rodents, the observed cleanliness of the housing facilities at the second sampling and the observed cleanliness of the individual pigs at both sampling time points.

Individual pigs that had received a prophylactic antibiotic group treatment had a higher likelihood of changing from *Chlamydiaceae* positive to negative or of remaining negative in their fecal swabs between the first and second sampling (Tables 2 and 4). Amoxicillin treatment did not reduce the chlamydial prevalence in fecal swabs, while treatment with C/CST as well as TSS had a reducing effect (Table 2). On herd level, however, the outcome was variable (Table 5): five herds showed an increase and four herds a decrease of chlamydial prevalence in conjunctival swabs; in fecal swabs, two herds showed an increase, five herds a decrease and in two herds the chlamydial prevalence remained the same. In farms without antibiotic group treatment (n = 18; farm nos. 1–5, 10–16, 18–21, 24, 25, 28), conjunctival positivity decreased in 10 farms, increased in seven and was unaltered in one farm. Fecal positivity was reduced in four farms, was increased in six farms and was unaltered in eight farms (S3 Table). Overall, the herd-based conjunctival prevalence was reduced by 6.5% in antibiotic group treated farms compared to 13.3% reduction in the untreated group. The fecal prevalence was reduced by 7.1% in the first group compared to a 0.5% rise in the untreated farms. The maximum reduction of 30% of fecal chlamydial burden was reached in farm 27 with tetracycline group treatment (Table 5).

**Table 5:** Difference of *Chlamydiaceae* prevalence in farms with antibiotic group treatment between the first and second sampling.

farm	group treatment ^a)^	conjunctival swab difference 1^st^ to 2^nd^ (%) ^§^	rectal swab difference 1^st^ to 2^nd^ (%) ^§^
**9**	CST	-80.5	-13.9
**17**	C	+20	±0
**22**	CST	+55	±0
**27**	CST	-10	-30
**29**	CST	+25	+5
**6**	TSS	-68.4	-5.3
**23**	TSS	+10	-15
**26**	TSS	+20	-10
**7**	A	-30	+5

^a)^ pro-/metaphylactic group treatment after 1st sampling, type of antimicrobial substance: A = amoxicillin; C = chlortetracycline; CST = chlortetracycline, sulfadimidin, tylosin; TSS = trimethoprime, sulfadimidin, sulfathiazole; 1^st^ = sampling at the beginning of the fattening period; 2^nd^ = sampling at the end of the fattening period

^§^ = significant difference between the individual farms.

Pigs in farms with single-pig antibiotic treatment during the fattening period showed no decrease in positivity.

The non-correlated significant influencing factors were included in a full regression model and are summarized in Table 6. Briefly, an independent association of the following factors and *Chlamydiaceae* positivity was found at the second sampling: all-in/all-out production, number of purchase farms, distance to ruminants, possible contact with wild boars and prophylactic antibiotic group treatment. The number of purchase farms and a prophylactic antibiotic group treatment were independently associated with fecal swabs that were positive for *Chlamydiaceae* at the first and negative at the second sampling. In *C*. *suis*, all-in/all-out production, number of purchase farms, distance to ruminants and possible contact with wild boars were independent influencing factors for positivity at the second time point. All-in/all-out production, number of purchase farms, distance to ruminants and possible contact with wild boars were independently associated with fecal swabs that were positive for *C*. *suis* at the first and negative at the second sampling. In *C*. *pecorum*, the results of the second sampling were independently influenced by distance to ruminants, possible contact with wild boars, sex and outdoor access.

**Table 6:** Results of multivariate analysis with step back procedure for infection risk. e: entered in full model (p ≤ 0.05 univariate) but eliminated in the final model;.: not entered in full model (p > 0.05 univariate); OR: odds ratio; CI: confidence interval.

Variable	Pigs positive in eye and/or fecal swab at 2^nd^ sampling OR (95% CI)			Fecal swabs shifted from positive to negative OR (95% CI)	
	*Chlamydiaceae*	*C*. *suis*	*C*. *pecorum*	*Chlamydiaceae*	*C*. *suis*
**All-in/ all-out**	3.5 (1.8–7.1)	1.6 (1.0–2.5)	.	.	0.5 (0.3–0.7)
**Number of purchase farms**	1.7 (1.3–2.4)	1.3 (1.0–1.5)	.	1.7 (1.1–2.6)	0.8 (0.6–0.95)
**Distance ruminants**	0.5 (0.4–0.7)	0.7 (0.6–0.9)	0.6 (0.5–0.8)	e	1.3 (1.1–1.6)
**Indirect contact with ruminants**	e	e	e	.	e
**Contact with wild boars**	4.1 (2.2–7.6)	2.4 (1.5–3.7)	2.8 (1.7–4.8)	.	0.4 (0.2–0.6)
**Antibiotic group treatment**	0.3 (0.1–0.6)	.	.	2.7 (1.2–5.9)	.
**Sex female**	.	.	1.8 (1.1–2.9)	.	.
**Outdoor access**	.	.	8.1 (2.8–23.6)	.	.

## Discussion

### *Chlamydiaceae* and *Chlamydia suis*

This is the first large-scale study to investigate *Chlamydiaceae* infections of fattening pigs at two time points in Switzerland. With a prevalence of 98.9%, almost all pigs in this study were positive for *Chlamydiaceae*. In the first sampling, 94.3% and in the second sampling 92.0% of the fecal swabs were positive for *Chlamydiaceae*, as well as 45.9 and 32.6% of the conjunctival swabs, respectively. Of those, *C*. *suis* was identified in 90.6% (first sampling) and 67.9% (second sampling) of fecal swabs and in 91.2% and 71.9% of the conjunctival samples. Thus, *C*. *suis* was by far the most common chlamydial species found in both rectal and conjunctival swabs in this study. *C*. *suis* has been previously found to be the most common *Chlamydia* sp. in pigs [37], being often found in the pig intestine [13,38,39], pig conjunctivae [32,40] and at a variety of other sites, such as in the male and female genital tract [7,41], nasal swabs [42], lung tissue [43] and the liver of aborted fetuses [44]. The high prevalence of *Chlamydiaceae* and *C*. *suis* in fecal swabs at both sampling time points in the present study was surprising. Englund et al. [30] found that 100% of the pigs (n = 36) from herds with diarrhea problems (n = 6) and 83% of the healthy control pigs (n = 12) from good performance herds (n = 4) tested positive for *Chlamydiaceae*; moreover, sequencing of selected ileal tissues revealed *C*. *suis*. In contrast, in the study of De Puysseleyr et al. [19], only 52% of rectal swabs from slaughter pigs in a Belgian slaughterhouse were tested positive with a *C*. *suis* species-specific PCR. Kauffold et al. [7] found 37.9% and 8.3% *C*. *suis* positive fecal samples in two different boar studs. Pollmann [39] tested 22 breeding sows three times with a *C*. *suis*-specific PCR and, compared to a single time sampling, the prevalence rose from 27% (single sampling) to 73% (three samplings), suggesting the possibility of intermittent fecal shedding. In the present study, the fecal swabs of most pigs remained positive during the study period and only a small proportion became negative or changed from negative to positive in both treated and untreated farms, which might also be explained by intermittent fecal shedding. In different hosts, *Chlamydia* sp. is able to inhabit the gastrointestinal tract over a long period of time, as recently reviewed by Rank and Yeruva [45]. The results of our study indicate that a similar long-term intestinal infection is common in pigs. Moreover, Pospischil et al. [46] showed that aberrant bodies of *C*. *suis*, the cryptic chlamydial form associated with persistence, also called the chlamydial stress response, can occur in the intestine of naturally and experimentally infected pigs.

In all herds, the prevalence at the first sampling time point was very high, which indicates that the pigs were already infected earlier or acquired the infection on the new farms after mixing with infected pigs. In the farrow-to-finish farms, mixing with foreign animals was not possible, although, the prevalence in these farms was as high as in other farms. The incubation period after chlamydial infection was determined by Guscetti et al. [47] in an intra-gastric inoculation model of 2–3 day-old gnotobiotic piglets, and first fecal shedding occurred as early as the second day after inoculation. Hence, early infection of *Chlamydia*-negative pigs by fecal-oral transmission through mixing with infected animals could have been possible before the first sampling time point, in particular in those herds where the study investigators performed the first sampling 48–72 hours after arrival of the pigs. Alternatively, piglets could have become infected prior to the fattening period.

The conjunctival prevalence of *Chlamydiaceae* (45.9% at the first and 32.6% at the second time point) was far below the fecal prevalence, but comparable to findings of a study by Becker et al. [32]. They found 42% positive Swiss pigs, but 89% positive pigs in Germany. Englund et al. [30] detected *Chlamydiaceae* in conjunctival swabs in Swedish pigs with conjunctivitis (82.8%) and without conjunctivitis (72.4%). Differences might be explained by different housing and management systems in these countries, which might influence predisposing factors and transmission routes. *Chlamydiaceae* shed through conjunctival discharge and feces can easily lead to horizontal transmission, auto- or reinfections. Moreover, contaminated dust can be the source of chlamydial infection, as indicated in our study, where *C*. *suis and C*. *pecorum* were detected in the dust of one farm and viable *C*. *suis* were also found in air samples of a Belgian pig slaughterhouse [19].

Regarding *Chlamydiaceae* copy numbers, fecal shedding was significantly higher than conjunctival shedding. This may be due to the fact that the DNA we found in the eyes does not necessarily represent the presence of viable *Chlamydia* but rather an eye contamination with chlamydial DNA fragments. This might also explain the short-term detection of some eye infections. The reasons for the rise in the mean copy number of positive conjunctival swabs at the second time point remained unclear. Nevertheless, it was still lower than fecal copy numbers indicating that the gut might represent a true site of active chlamydial replication. An explanation for the lower mean of copy numbers in positive fecal swabs at the second time point could be due to stable living conditions of the animals for at least three months, in contrast to the situation at the first sampling time point after introduction into a new farm. The hypothetical time point of the initial infection is thereby also months ago and in the meantime the host organism may have adapted itself leading to a more balanced gut microbiota and reduced replication.

### Other *Chlamydia* spp.

*C*. *pecorum* was frequently found in fecal swabs and sporadically in conjunctival swabs, especially as a co-infection with *C*. *suis*. In other studies and case reports, *C*. *pecorum* was not detected in conjunctival swabs [26,32,48] and rarely in semen and fecal samples of boars [7] or as a mixed infection with *C*. *suis* in aborted material [44]. Ruminants are the main host for *C*. *pecorum* and subclinical infections are common [49]. All pigs in our study were surrounded by ruminants at different distances and contact opportunities. We were able to show a high correlation between potential direct or indirect contact between ruminants and pigs and *C*. *pecorum* positive samples. The high likelihood of contact with ruminants in our study compared to other pig husbandry systems could explain the relatively high prevalences. Interestingly, all eye infections at the first time point were no longer present at the second time point in the same individuals. This may be due to intermittent shedding or fast clearance of conjunctival infections.

As it was only detected once in a conjunctival swab, *C*. *abortus* was not an important *Chlamydia* sp. in this study. Comparable to *C*. *pecorum*, the *C*. *abortus* infection was no longer present at the second sampling. A possible infection source in the single positive case was a sheep flock with potential direct contact with the pig herd. *C*. *abortus* was found by other investigators in the cervical swabs of breeding sows [4], conjunctival swabs of sows and semen of boars [48], lung and intestine of healthy slaughter pigs and pigs with respiratory disease [50], and in an aborted fetus [44].

*C*. *psittaci* was not detected in any of the pigs, despite possible contact with wild birds and poultry in the surroundings of most pig herds. This contrasts with the report of Vanrompay et al. [5], where a chlamydial strain was found in a Belgian pig, which was highly related to pigeon *C*. *psittaci* serovar B strains; thus a potential transmission from birds to pigs was assumed. While *C*. *abortus* and *C*. *psittaci* are known zoonotic pathogens, the zoonotic potential of *C*. *suis* and *C*. *pecorum* is still a matter of debate [18–20,51]. All tested conjunctival swabs of farmers in this study were negative, thus a zoonotic transmission could not be detected. However, the human population tested was very small and, apart from the eyes, no other body sites were tested.

### Clinical symptoms and influencing factors

It has been shown that *C*. *suis* is not a primary pathogen when colonizing pig intestines during natural infection [2,52] but could cause intestinal lesions and diarrhea after experimental oral or intragastric infection of piglets [47,53]. *C*. *pecorum* has been associated with pneumonia, polyarthritis, pleuritis, pericarditis and abortion in pigs [54]. In this study, a correlation between diarrhea and fecal swab positivity for *C*. *suis* and *C*. *pecorum* could be found. In some farms, typical diarrhea-causative agents were identified, but a bacteriological examination was not performed in all affected farms. It can be assumed that *C*. *suis* was acting as a facultative pathogen, as reported previously in co-infections with primary pathogenic agents like *Salmonella* [48,55]. On the other hand, there was no clear association between pneumonia and chlamydial infection, which is in accordance with the studies of Reinhold et al. [56,57]. These authors showed that in contrast to experimental infection, naturally acquired *Chlamydiaceae* infections do not cause respiratory symptoms in pigs.

In accordance with Englund et al. [30] and Polkinghorne et al. [31] no correlation between conjunctivitis and chlamydial eye infections was found in this study. In contrast, Becker et al. [32] found a significant correlation between conjunctivitis and chlamydial positivity in Swiss but not in German pigs. While they attributed the difference between Swiss and German pigs to the contrast between intensive and extensive farming, this influencing factor cannot be explanatory here, because all farms belonged, roughly speaking, to an extensive management system. Ocular experimental infections with an ocular swine *C*. *trachomatis*-like strain H7 [58] showed that the ocular infection remained subclinical in gnotobiotic piglets, and that lesions were only visible by histological examination of the conjunctiva and nictitating membrane. In the present study, conjunctival positivity declined from the first to the second time point, maybe due to increasing immunity, while the conjunctivitis symptoms increased. Hence, it can be assumed that the clinical symptoms were due to other, most probably environmentally related, factors such as a high concentration of dust, ammonia, hydrogen sulfide, and other decomposition gases [59]. The occurrence of other conjunctivitis-causing infectious agents is also conceivable, e.g. *Mycoplasma* spp., porcine cytomegalovirus and swine influenza virus, but they were not examined. Although chlamydiae do not seem to cause the clinical symptoms, they may predispose to conjunctivitis. Older literature even reports that the conjunctivae of pigs are moderately reddened physiologically [60]. Apart from that, the study investigators often had the impression that iatrogenic stress during sampling led to reddening of the conjunctivae due to elevated blood pressure. Becker et al. [32] reported a rate of conjunctivitis in Swiss pigs (37.3%) similar to that in this study at the second sampling time point.

When comparing single pig results at the second sampling, a few management factors influenced the rate. First of all, farrow-to-finish farms and those with continuous production had a lower risk of being infected than others. However, an increasing number of different pig origins raised the risk of *Chlamydiaceae* infection. In farms where self recruitment is not carried out and fattening pigs are moved in at approximately three months of age, the pigs have to cope with many environmental changes in the new housing facility as well as the microorganisms present in the new housings and in the pigs from foreign farms. Therefore the risk of being infected with any bacteria is increased. In addition, the transport and unfamiliar situation in a new pen with unknown group members puts stress on the pigs, which, in turn, usually enhances the intestinal growth of pathogens or the likelihood of their shedding due to immune suppression [61,62]. Based on the concept of persistence, stress could also reactivate a chlamydial infection [48].

Interestingly, increased hygiene management (as reported by the farmer) could not lower *Chlamydia* positivity. This means that the type and frequency of the cleaning procedure and use of disinfectants did not influence chlamydial infection. However, the information on hygiene management provided by the farmer was possibly not entirely reliable because of his subjective evaluation. The actual cleanliness of the housing was also evaluated by the study investigators. At the first sampling time point, good housing hygiene had a reducing influence on the detection rate of *Chlamydiaceae*. On the other hand, this connection could not be made at the second sampling time point or for individual animal-surface cleanliness. In summary, only two individual time points could be assessed limiting the significance of this observation.

Swiss pig farms are usually not double-fenced and are situated in rural areas by law; therefore contact with wild boars or other wild mammals is possible in farms with outdoor areas. We found a clear association between farms with potential wild boar contact and the risk of infection. This is an important finding, because other more fatal agents could potentially be transmitted in this way, as wild boars represent a reservoir for several pathogens [63]. Having unprotected outdoor areas also enables contact with pastured ruminants and wild birds. Free-ranging ruminants in Switzerland have been shown to sporadically harbor *Chlamydiaceae* [64].

In *C*. *pecorum* infections, there was a link between the presence of bedding material and positivity at the second sampling time point. This may be a confounding factor, because these farms also mostly had outdoor access, and this also increased the infectivity rate. However, this association was not present in infections with *Chlamydiaceae* and *C*. *suis*. EBs of *Chlamydia* sp. are reported to survive in dry feces, dust or litter for several months [49] and contaminated bedding material could consequently represent a source of infection.

Prophylactic antibiotic treatment was applied in nine herds, usually as routine procedure in farms with pigs from different origins to prevent common grower pig health issues like intestinal tract and respiratory diseases, and to improve growth performance. A therapeutic effect on chlamydiae can be expected from tetracyclines as well as sulfonamide and trimethoprim combinations [65]. These and amoxicillin were used for group treatments in the investigated farms. Amoxicilllin is a derivate of penicillin, which is known to induce chlamydial persistence *in vitro* instead of killing the RBs [45]. In this study, we could not retrieve information about any treatment before the beginning of the fattening period. Only in one farrow-to-finish farm (no. 28), did we know about the administration of chlortetracycline, sulfadimidine and tylosin after weaning and prior to our first sampling. The rate of positive conjunctival (50%) and fecal samples (90%) in this particular farm at the first sampling time point was comparable to that in the untreated farms. Five farms in this study treated the pig herd after the first sampling with an oral medication containing chlortetracycline. Four of them used a combination product with tylosin, which belongs to the protein synthesis inhibiting macrolides. Azithromycin also belongs to this group and is usually the drug of choice to treat human chlamydial infections. Nevertheless, the *Chlamydiaceae* prevalence in conjunctival and fecal samples only decreased in two farms, whereas in the other farms, the prevalences remained unchanged or even increased (Table 5). In the TSS treated farms, the reduction of positivity in conjunctival and fecal swabs was higher than in the untreated farms and also higher than in the tetracycline/tylosin-group. One farm (no. 7) administered Amoxicillin as an oral group treatment, because the pigs developed respiratory symptoms. All conjunctival samples that were positive at the beginning were negative at the second time point, but fecal positivity increased slightly. None of the individually antimicrobial treated pigs became negative for *Chlamydiaceae* at the second time point.

In summary, none of the antibiotic treatments in this study was able to clear the chlamydial infections on herd level despite individual pigs becoming negative by the end of the fattening period. The tetracycline resistance of *C*. *suis* is well known, while resistance to sulfonamide and trimethoprim combinations was described once [23]. Our observations match those of a study of Reinhold et al. [42], in which the short-term treatment with enrofloxacin, a fluoroquinolone, resulted in a recurrence and increased quantity of *Chlamydia* spp. in fecal and nasal swabs after initial reduction. It is possible that the treatment duration in the farms of this study was too short to clear the infection; it was administered for five to twelve days. Moreover, Yeruva et al. [66,67] showed that the gut is a site of persistence and source of possible reinfection in a mouse model. This is facilitated by an immune down-regulating effect in the intestine, resulting in the inability of the host to resolve the infection. The same group also proved a reduced susceptibility of the gut compared to the genital tract to azithromycin treatment.

In conclusions, the recommendation of antibiotic treatment in pig chlamydiosis should be reconsidered regarding its necessity and effectivity. Apart from that, the efficacy of prophylactic or metaphylactic use of oral group treatment with antibiotics in pig farms in light of the highly prevalent intestinal chlamydial infections should be critically reviewed. The clinical impact of chlamydial infection seems to be of low significance in regard to conjunctivitis but they may contribute to diarrhea. Therefore, routine diagnostics of herd-based diarrhea problems should include *Chlamydiaceae* testing. A follow-up examination will investigate the pathogenicity and antibiotic susceptibility of *C*. *suis* found in this study.

## Supporting Information

S1 Table Details of farms investigated in this study. (DOCX)

S2 Table Content of questionnaire used to survey farmers. (DOCX)

S3 Table Results of *Chlamydiaceae* screening of fattening pigs from 29 farms. (DOCX)

S1 Dataset Listing of all samples with relevant herd-based information, PCR and Arraymate microarray results. (XLSX)
