# Supplementary material for: Prevalence of Chlamydial Infections in Fattening Pigs and Their Influencing Factors
Source: PLoS One. 2015 Nov 30;10(11):e0143576. doi: 10.1371/journal.pone.0143576 (PMC4664257; doi:10.1371/journal.pone.0143576)
Supplement: S1 Table — (DOCX) [file pone.0143576.s001.docx]

**S1 Table.** **Details of farms investigated in this study.** 1^st^ = sampled at the beginning of the fattening period; 2^nd^ = sampled at the end of the fattening period; na = not available; N = no; Y = yes; ^a)^ pro-/metaphylactic group treatment after 1st sampling, type of antimicrobial substance: A = amoxicilllin; C = chlortetracycline; CST = chlortetracycline, sulfadimidine, tylosin; TSS = trimethoprime, sulfadimidine, sulfathiazole; * = CST was administered after weaning but before the 1^st^ sampling

^b)^ class of applied therapeutic antimicrobial substance during fattening period: AG = aminoglycoside; B = β-lactam antibiotic; C = cephalosporin; F = fluoroquinolone; TET = tetracycline; route of administration: ^t^ = topic treatment route; all others: intramuscular injection; ^c)^ direct: snout contact between animals possible; indirect: transmission via non-living vectors possible

| **farm** | **No. of fattening pigs** | | | **female pigs (%)** | **Antibiotic treatment** | | **Outdoor access** | **Contact with ruminants ^c)^** | |
| --- | --- | --- | --- | --- | --- | --- | --- | --- | --- |
|  | total | 1^st^ | 2^nd^ |  | group ^a)^ | single pig ^b)^ |  | direct | indirect |
| **1** | 70 | 70 | 70 | 53 | - | TET^t^, B | N | N | Cattle |
| **2** | 50 | 20 | 20 | 40 | - | - | N | N | Cattle |
| **3** | 600 | 20 | 20 | 55 | - | B | N | N | N |
| **4** | 496 | 20 | 20 | 75 | - | B, AG | Y | N | N |
| **5** | 60 | 10 | 9 | 40 | - | - | N | N | Cattle |
| **6** | 108 | 20 | 19 | 45 | TSS | B | Y | Cattle | Cattle |
| **7** | 115 | 20 | 20 | 45 | A | B | Y | N | Cattle |
| **8** | 220 | 20 | na | 65 | na | na | Y | N | Cattle |
| **9** | 200 | 36 | 36 | 47 | CST | - | Y | N | Cattle, Goats |
| **10** | 170 | 20 | na | 45 | - | na | N | N | N |
| **11** | 245 | 20 | 19 | 55 | - | TET, B, AG, F, C | Y | N | Cattle, Goats |
| **12** | 60 | 20 | 19 | 50 | - | TET^t^, C | N | N | N |
| **13** | 180 | 20 | 19 | 50 | - | TET | Y | N | Cattle, Sheep |
| **14** | 320 | 20 | 18 | 55 | - | B, AG | Y | N | N |
| **15** | 200 | 20 | 20 | 50 | - | - | Y | Sheep | Sheep |
| **16** | 160 | 20 | 20 | 65 | - | - | Y | Cattle | Cattle, Goats |
| **17** | 441 | 20 | 20 | 40 | C | TET, B, AG, M | Y | N | N |
| **18** | 250 | 20 | 20 | 100 | - | - | N | N | N |
| **19** | 800 | 20 | 20 | 60 | - | TET, B, AG | Y | N | Cattle |
| **20** | 260 | 20 | 20 | 70 | - | - | Y | Cattle | Cattle |
| **21** | 341 | 20 | 20 | 40 | - | - | N | N | N |
| **22** | 210 | 20 | 20 | 50 | CST | - | Y | N | Cattle |
| **23** | 239 | 20 | 20 | 55 | TSS | B, AG | Y | N | Cattle |
| **24** | 228 | 20 | 20 | 30 | - | - | Y | N | N |
| **25** | 272 | 20 | 20 | 100 | - | - | N | N | N |
| **26** | 475 | 20 | 20 | 50 | TSS | B | Y | Sheep | Cattle, Sheep |
| **27** | 1000 | 20 | 20 | 35 | CST | B | N | N | N |
| **28** | 176 | 20 | 20 | 75 | * | B | Y | N | Cattle |
| **29** | 900 | 20 | 20 | 50 | CST | - | Y | N | N |
| **total** | **8846** | **636** | **589** | **55** |  |  |  |  |  |
