# Supplementary material for: Prevalence of Chlamydial Infections in Fattening Pigs and Their Influencing Factors
Source: PLoS One. 2015 Nov 30;10(11):e0143576. doi: 10.1371/journal.pone.0143576 (PMC4664257; doi:10.1371/journal.pone.0143576)
Supplement: S2 Table — (DOCX) [file pone.0143576.s002.docx]

**S2 Table. Content of questionnaire used to survey farmers.**

| Subject | Factors |
| --- | --- |
| Herd Type | Type of farm and animals (breeding sows, fattening pigs), numbers of animals |
| Housing | Bedding material, possibility of direct contact between pig pens, access to outdoor area |
| Management practice | Recruitment of animals, number of suppliers, all-in/all-out production, separation of diseased or slow growing animals |
| Biosecurity | Shared use of devices and vehicles between housings and/or farms, hygiene gate to pig housing, level of insect and rodent infestation and pest control, other livestock and pets on farm and distance to pigs, contact with other domestic or wild animals, other livestock within 1000 m |
| Cleaning and disinfection procedures | Cleaning frequency, high-pressure cleaning, temperature of cleaning water, use of cleaning agents, disinfection after cleaning |
| Health | Previous detection of pathogens, dose and duration of antimicrobial treatment and other medical treatment, health problems in herds |
