# Supplementary material for: Prevalence of Chlamydial Infections in Fattening Pigs and Their Influencing Factors
Source: PLoS One. 2015 Nov 30;10(11):e0143576. doi: 10.1371/journal.pone.0143576 (PMC4664257; doi:10.1371/journal.pone.0143576)
Supplement: S3 Table — (DOCX) [file pone.0143576.s003.docx]

**S3 Table.** **Results of *Chlamydiaceae* screening of fattening pigs from 29 farms.** 1^st^ sampling = sampling at the beginning of the fattening period; 2^nd^ sampling = sampling at the end of the fattening period; na = not available.

|  | **conjunctival swab** | | | **fecal swab** | | |
| --- | --- | --- | --- | --- | --- | --- |
| **farm** | No. of pigs positive at 1^st^ sampling (%) | No. of pigs positive at 2^nd^ sampling (%) | difference 1^st^ to 2^nd^ (%) | No. of.pigs positive at 1^st^ sampling (%) | No. of.pigs positive at 2^nd^ sampling (%) | difference 1^st^ to 2^nd^ (%) |
| **1** | 24/70 (**34.3**) | 37/70 (**52.9**) | +18.6 | 67/70 (**95.7**) | 70/70 (**100**) | +4.3 |
| **2** | 3/20 (**15**) | 8/20 (**40**) | +25 | 20/20 (**100**) | 20/20 (**100**) | ±0 |
| **3** | 14/20 (**70**) | 15/20 (**75**) | +5 | 19/20 (**95**) | 20/20 (**100**) | +5 |
| **4** | 20/20 (**100**) | 17/20 (**85**) | -15 | 19/20 (**95**) | 20/20 (**100**) | +5 |
| **5** | 2/10 (**20**) | 3/9 (**33.3**) | +13.3 | 10/10 (**100**) | 9/9 (**100**) | ±0 |
| **6** | 20/20 (**100**) | 6/19 (**31.6**) | -68.4 | 20/20 (**100**) | 18/19 (**94.7**) | -5.3 |
| **7** | 6/20 (**30**) | 0/20 (**0**) | -30 | 18/20 (**90**) | 19/20 (**95**) | +5 |
| **8** | 15/20 (**75**) | na | na | 20/20 (**100**) | na | na |
| **9** | 30/36 (**83.3**) | 1/36 (**2.8**) | -80.5 | 36/36 (**100**) | 31/36 (**86.1**) | -13.9 |
| **10** | 19/20 (**95**) | na | na | 20/20 (**100**) | na | na |
| **11** | 5/20 (**25**) | 1/19 (**5.3**) | -20 | 20/20 (**100**) | 19/19 (**100**) | ±0 |
| **12** | 11/20 (**55**) | 0/19 (**0**) | -55 | 19/20 (**95**) | 17/19 (**89.5**) | -5.5 |
| **13** | 18/20 (**90**) | 0/19 (**0**) | -90 | 20/20 (**100**) | 18/19 (**94.7**) | -5.3 |
| **14** | 20/20 **(100)** | 6/18 (**33.3**) | -66.7 | 20/20 (**100**) | 18/18 (**100**) | ±0 |
| **15** | 8/20 (**40**) | 8/20 (**40**) | ±0 | 19/20 (**95**) | 19/20 (**95**) | ±0 |
| **16** | 16/20 (**80**) | 13/20 (**65**) | -15 | 19/20 (**95**) | 20/20 (**100**) | +5 |
| **17** | 3/20 (**15**) | 7/20 (**35**) | +20 | 20/20 (**100**) | 20/20 (**100**) | ±0 |
| **18** | 1/20 (**5**) | 4/20 (**20**) | +15 | 19/20 (**95**) | 18/20 (**90**) | -5 |
| **19** | 17/20 (**85**) | 8/20 (**40**) | -45 | 20/20 (**100**) | 20/20 (**100**) | ±0 |
| **20** | 7/20 (**35**) | 8/20 (**40**) | +5 | 19/20 (**95**) | 19/20 (**95**) | ±0 |
| **21** | 4/20 **(20)** | 0/20 **(0)** | -20 | 18/20 **(90)** | 16/20 **(80)** | -10 |
| **22** | 0/20 **(0)** | 11/20 **(55)** | +55 | 12/20 **(60)** | 12/20 **(60)** | ±0 |
| **23** | 0/20 **(0)** | 2/20 **(10)** | +10 | 17/20 **(85)** | 14/20 **(70)** | -15 |
| **24** | 1/20 **(5)** | 12/20 **(60)** | +55 | 15/20 **(75)** | 16/20 **(80)** | +5 |
| **25** | 6/20 **(30)** | 4/20 **(20)** | -10 | 20/20 **(100)** | 20/20 **(100)** | ±0 |
| **26** | 4/20 **(20)** | 8/20 **(40)** | +20 | 20/20 **(100)** | 18/20 **(90)** | -10 |
| **27** | 4/20 **(20)** | 2/20 **(10)** | -10 | 17/20 **(85)** | 11/20 **(55)** | -30 |
| **28** | 10/20 **(50)** | 2/20 **(10)** | -40 | 18/20 **(90)** | 20/20 **(100)** | +10 |
| **29** | 4/20 **(20)** | 9/20 **(45)** | +25 | 19/20 **(95)** | 20/20 **(100)** | +5 |
| **total** | **292/636 (45.9)** | **192/589 (32.6)** | **-13.3** | **600/636 (94.3)** | **542/589 (92.0)** | **-2.3** |
